# Sol-Gel Synthesis of Silicon-Doped Lithium Manganese Oxide with Enhanced Reversible Capacity and Cycling Stability

**DOI:** 10.3390/ma11081455

**Published:** 2018-08-15

**Authors:** Hongyuan Zhao, Dongdong Li, Yashuang Wang, Fang Li, Guifang Wang, Tingting Wu, Zhankui Wang, Yongfeng Li, Jianxiu Su

**Affiliations:** 1 School of Mechanical & Electrical Engineering, Henan Institute of Science and Technology, Xinxiang 453003, China; wtingtingwu@163.com (T.W.); luckywzk@126.com (Z.W.); yongfengli121@outlook.com (Y.L.); 2 Research Branch of Advanced Materials & Green Energy, Henan Institute of Science and Technology, Xinxiang 453003, China; Lidongdong1994@126.com (D.L.); yashuangwang1102@126.com (Y.W.); lifang@hist.edu.cn (F.L.); 3 School of Resources, Environment and Materials, Guangxi University, Nanning 530004, China

**Keywords:** lithium-ion batteries, LiMn_2_O_4_, sol-gel method, Si-doping, electrochemical properties

## Abstract

A series of silicon-doped lithium manganese oxides were obtained via a sol-gel process. XRD characterization results indicate that the silicon-doped samples retain the spinel structure of LiMn_2_O_4_. Electrochemical tests show that introducing silicon ions into the spinel structure can have a great effect on reversible capacity and cycling stability. When cycled at 0.5 C, the optimal Si-doped LiMn_2_O_4_ can exhibit a pretty high initial capacity of 140.8 mAh g^−1^ with excellent retention of 91.1% after 100 cycles, which is higher than that of the LiMn_2_O_4_, LiMn_1.975_Si_0.025_O_4_, and LiMn_1.925_Si_0.075_O_4_ samples. Moreover, the optimal Si-doped LiMn_2_O_4_ can exhibit 88.3 mAh g^−1^ with satisfactory cycling performance at 10 C. These satisfactory results are mainly contributed by the more regular and increased MnO_6_ octahedra and even size distribution in the silicon-doped samples obtained by sol-gel technology.

## 1. Introduction

As green energy, the application of lithium-ion batteries has been extended to various fields in our life [1,2,3]. At present, an increasing number of countries are publishing timetables and road maps for forbidding the sale of traditional fuel vehicles. Against this backdrop, the research and development of lithium-ion batteries is receiving more and more attention at home and abroad. It is generally known that the cathode materials greatly influence the electrochemical performance of lithium-ion batteries. Among appropriate cathode materials, LiMn_2_O_4_ possesses the distinct advantages of low price, mature production technology and non-pollution characteristic, and is conducive to sustainable development and large-scale application [4,5]. However, it is a great pity that the poor cycling life cannot satisfy the needs of commercial application of LiMn_2_O_4_. This unwelcome fact is related to Jahn-Teller distortion, manganese dissolution and non-uniform particle-size distribution [6,7,8].

Until now, a large number of optimization strategies have been developed to enhance the electrochemical performance of LiMn_2_O_4_ [7,9,10,11,12,13,14]. According to the reported works [7,15], the surface coating treatment can improve the cycling performance to some degree by inhibiting the dissolution of manganese in the electrolyte. Unfortunately, this strategy cannot fundamentally reduce the negative impacts of the Jahn-Teller distortion effect, and also decreases the discharge capacity [12]. These facts indicate that surface modification is not a top-priority optimization method to enhance the comprehensive performance of LiMn_2_O_4_. Therefore, lots of researchers choose to use the doping strategy to avoid the shortcoming of the surface coating treatment [11,16,17]. Yu et al. [17] prepared Li_1+x_Mn_2−x_O_4_ samples by a solid-state sintering method. The obtained Li_1.06_Mn_1.94_O_4_ sample presents better cycling performance because the introduction of lithium ions can weaken the ordering of lithium ions and enhance the structure stability. Xu et al. [18] reported the synthesis of LiZn_x_Mn_2−x_O_4_ by a solution combustion method. The research results showed that Zn-doping can enhance the cycling performance by reducing the negative impacts of the Jahn-Teller distortion effect. Furthermore, the LiAl_x_Mn_2−x_O_4_ samples synthesized by solution combustion technique present better cycling life, which benefits from the effective inhibition of the Jahn-Teller distortion by Al-doping [19]. These analyses indicate that introducing other cations can actually enhance the cycling life of LiMn_2_O_4_. It should be noted, however, that introducing some monovalent cations, bivalent cations or trivalent cations can produce certain negative effects on reversible capacity because of the decrease of Mn^3+^ ions, which has previously been confirmed [11,20,21,22]. Based on all of the above studies, the introduction of some tetravalent cations has been proposed to effectively enhance the electrochemical performance of LiMn_2_O_4_ because this modification strategy can avoid the decrease of Mn^3+^ ions [23,24].

Herein, we have successfully obtained a series of silicon-doped lithium manganese oxides (LiMn_2−x_Si_x_O_4_, x ≤ 0.10) by sol-gel technology. The effect of silicon doping content on the structures, morphologies and electrochemical properties of the LiMn_2−x_Si_x_O_4_ samples obtained by sol-gel technology is discussed. The results indicate that the optimal silicon-doped sample prepared by sol-gel technology shows pretty high reversible capacity and outstanding cycling life.

## 2. Materials and Methods

The silicon-doped lithium manganese oxides (LiMn_2−x_Si_x_O_4_, x ≤ 0.10) were obtained via a sol-gel process with tetraethoxysilane (TEOS, Sinopharm Chemical Reagent Co., Ltd., Shanghai, China) as the dopant. Firstly, stoichiometric lithium hydroxide (0.8812 g) and citric acid (4.4129 g) were dissolved in deionized water (20 mL). Under vigorous stirring, the manganese acetate solution (1.5 M) and mixed solution of TEOS (0.2083 g) and ethanol solution (3.0 mL) were added dropwise into the above-mentioned solution at 50 °C. Then, a certain amount of NH_3_·H_2_O (Sinopharm Chemical Reagent Co., Ltd., Shanghai, China) was added dropwise into the mixed solution to adjust the pH value to 7–8, and the temperature was adjusted to 70 °C. After continuous stirring for a few hours, a reddish-brown sol was formed, which was then dried at 110 °C. The obtained dried gel was sintered at 450 °C for 4 h and then further sintered at 750 °C for 18 h at a heating and cooling speed of 5 °C·min^−1^. To investigate the influence of the Si-doping, an undoped LiMn_2_O_4_ spinel was prepared under the same conditions.

The crystal structures of the obtained silicon-doped LiMn_2_O_4_ samples were studied by X-ray diffraction technique (XRD, Bruker DX-1000, Karlsruhe, Germany) with Cu Kα radiation (λ = 0.15406 nm). Both transmission electron microscopy (TEM, JEOL JEM-3010, Tokyo, Japan) and scanning electron microscopy (SEM, JEOL JSM-6360LV, Tokyo, Japan) analytical techniques were used to study the surface morphologies and microstructures.

The active electrode consisted of the obtained silicon-doped LiMn_2_O_4_ samples, conductive acetylene black and polyvinylidene fluoride (Weight Ratio = 85:10:5). The anode material and diaphragm were lithium foil and Celgard 2400 polymer (Charlotte, NA, USA), respectively. The mixture of 1 M LiPF_6_, ethyl methyl carbonate (EMC), ethylene carbonate (EC) and dimethyl carbonate (DMC) was used as electrolyte (EMC: EC: DMC = 1:1:1) (Guangzhou Tinci Materials Technology Co., Ltd., Guagnzhou, China). The electrochemical measurement was executed on NEWARE battery testing system. The cyclic voltammogram results and electrochemical impedance spectroscopy (EIS) were tested by CS-350 electrochemical workstation (Wuhan Corrtest Instruments Crop., Ltd., Wuhan, China).

## 3. Results and Discussion

**Figure 1:**
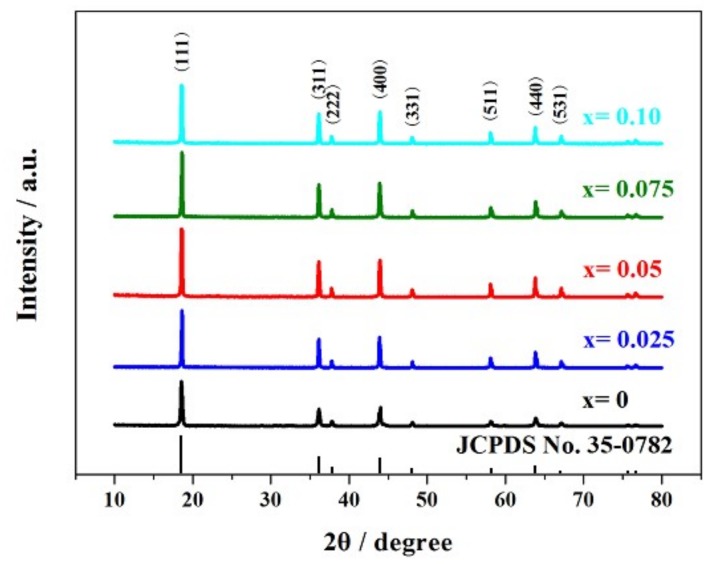
XRD patterns of the LiSi_x_Mn_2−x_O_4_ (x = 0, 0.025, 0.05, 0.075 and 0.10) samples.

To investigate the influence of Si-doping content on the crystalline phase of LiMn_2_O_4_, XRD was performed on the obtained samples. As shown in Figure 1, the characteristic diffraction peaks of all the Li_Mn2−x_Si_x_O_4_ samples obtained by sol-gel technology agree with that of LiMn_2_O_4_ (JCPDS No. 35-0782), suggesting the silicon-doped lithium manganese oxides obtained by sol-gel technology possess the cubic spinel structure, with lithium and manganese ions located at tetrahedral sites (8a) and octahedral sites (16d), respectively [25]. In addition, the (220) characteristic diffraction peak will be observed if the tetrahedral sites are occupied by other cations [26]. However, note that the (220) peak does not appear in the XRD patterns of the silicon-doped LiMn_2_O_4_ samples, indicating the substitution of silicon ions for manganese ions.

Table 1 presents the relevant parameters of the LiMn_2−x_Si_x_O_4_ samples obtained by sol-gel technology. It is clear from the data that all the LiMn_2−x_Si_x_O_4_ samples belong to Fd-3m space group. As the silicon doping content increases, the lattice parameter of these samples gradually increases. According to the reported results [24], the silicon-doped spinel presents longer Mn−O bond length and larger MnO_6_ octahedra. Moreover, the O−Mn−O angle in the Si-doped spinel presents values closer to 90. These results suggest that introducing some silicon ions leads to the more regular and increased MnO_6_ octahedra, which could explain the increase of lattice parameter and cell volume. In addition, Si-doping showed a great influence on the (400) FWHM value and the (311)/(400) intensity ratio. Among all the silicon-doped spinels, the LiMn_1.95_Si_0.05_O_4_ sample shows a smaller (400) FWHM value and (311)/(400) intensity ratio than the other samples, which is consistent with the results of previous research [27], suggesting higher crystallinity and longer cycling life.

**Figure 2:**
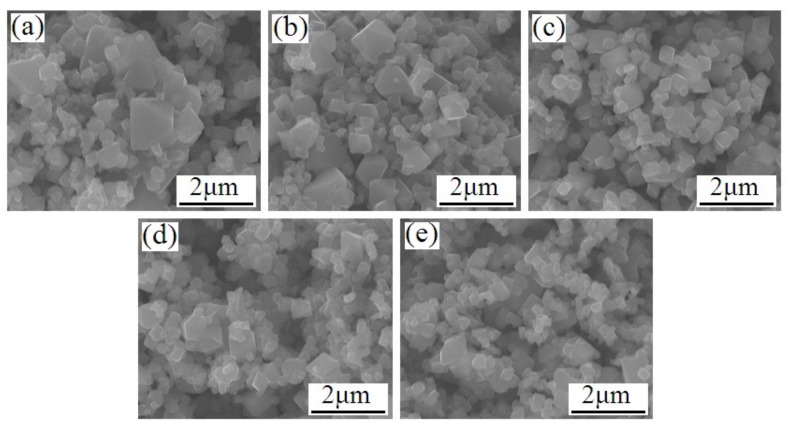
SEM images of the LiSi_x_Mn_2−x_O_4_ samples: (**a**) x = 0, (**b**) x = 0.025, (**c**) x = 0.05, (**d**) x = 0.075, (**e**) x = 0.10.

**Figure 3:**
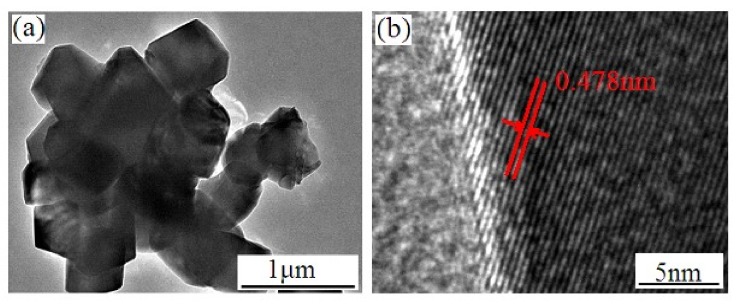
(**a**) TEM image and (**b**) HRTEM image of the LiSi_0.05_Mn_1.95_O_4_ sample.

The SEM images of the LiMn_2−x_Si_x_O_4_ samples are shown in Figure 2. The undoped LiMn_2_O_4_ particles shown in Figure 2a present an uneven size distribution. The corresponding range of particle size is from 0.1 to 1.6 μm. For the Si-doped LiMn_2_O_4_, the introduction of some silicon ions can optimize the mean diameter and size distribution. When the silicon doping content increases, the mean diameter of the LiMn_2−x_Si_x_O_4_ (0.025 ≤ x ≤ 0.10) has a decreasing tendency, which may be interpreted as the nucleation rate of silicon-doped samples exceeding the growth of particles with the silicon doping [5,28]. In particular, the LiMn_1.95_Si_0.05_O_4_ particles shown in Figure 2c present the most uniform size distribution, which is conducive to the enhancement of cycling life [28,29]. The above-mentioned results suggest that introducing some silicon ions can effectively improve the crystallinity and optimize the size distribution. Figure 3a,b shows the TEM and HRTEM images of the representative LiMn_1.95_Si_0.05_O_4_ sample. It can be observed that the growth of sample particles matches the (111) direction, and the lattice fringes of 0.478 nm correspond to the spinel lattice structure [30].

**Figure 4:**
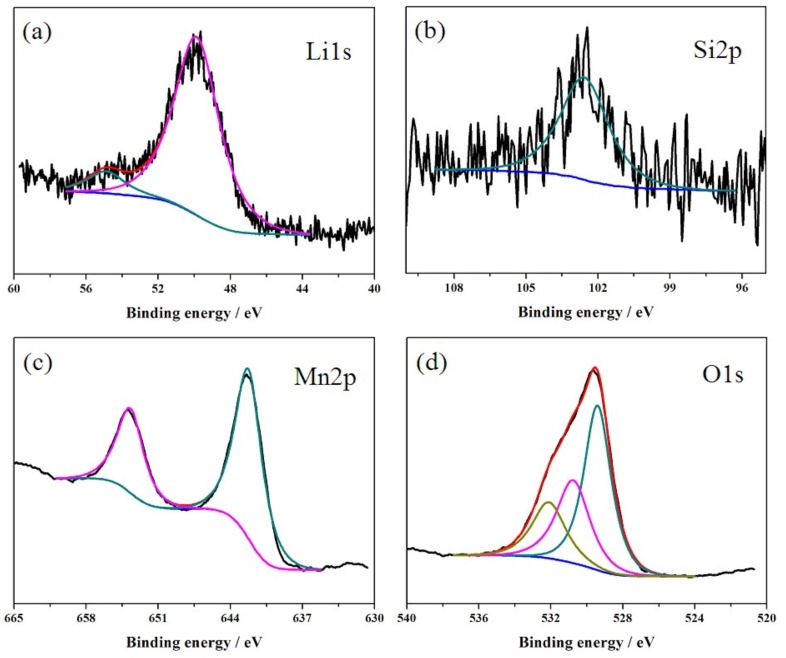
XPS spectra of Li1s (**a**), Si2p (**b**), Mn2p (**c**) and O1s (**d**) in the LiSi_0.05_Mn_1.95_O_4_ sample.

The XPS spectra of Li1s, Si2p, Mn2p and O1s in the LiMn_1.95_Si_0.05_O_4_ sample are shown in Figure 4. According to these results, we can obtain information regarding the chemical and electronic state. As shown in Figure 4a,c–d, the oxidation states of Li1s, Mn2p and O1s can be inferred from the binding energy peaks, which are consistent with the existing results [31]. it is important to note that the binding energies of Mn2p_3/2_ correspond to the Mn^3+^ ions (641.7 eV) and Mn^4+^ ions (643.1 eV), respectively [32]. However, the Mn2p_3/2_ binding energy shown in Figure 4c is at 642.6 eV, suggesting the mixture situation of Mn^3+^ and Mn^4+^ in the silicon-doped sample obtained by sol-gel technology. Figure 4b presents the XPS spectra of Si2p. We can deduce that the corresponding oxidation state is at 102.1 eV, which is in good agreement with the reported results [24].

**Figure 5:**
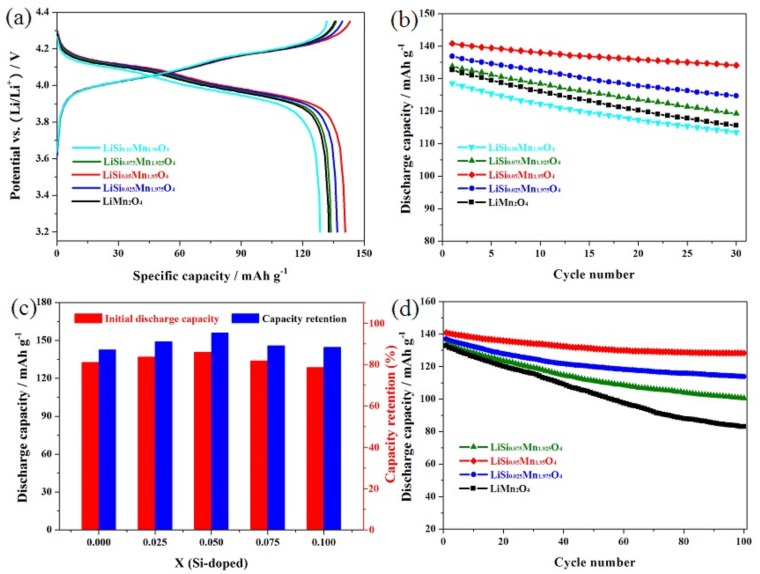
(**a**) Initial charge-discharge curves and (**b**) Cycling performance of the LiSi_x_Mn_2−x_O_4_ (x = 0, 0.025, 0.05, 0.075 and 0.10) samples; (**c**) Comparison plots of the initial discharge capacities and capacity retentions; (**d**) Long Cycling performance of the LiSi_x_Mn_2−x_O_4_ (x = 0, 0.025, 0.05, 0.075) samples.

Figure 5a presents the first discharge curves of the LiMn_2−x_Si_x_O_4_ (x = 0, 0.025, 0.05, 0.075 and 0.10) samples. All these silicon-doped samples present characteristic discharge curves, showing two distinct voltage platforms around 4.10–4.15 V and 3.95–4.00 V, suggesting that introducing silicon ions did not change the electrochemical redox reaction mechanism, and that all these Si-doped LiMn_2_O_4_ sample processes comprise two extraction/insertion processes of lithium ions [33]. Figure 5b presents the cycling life of the LiMn_2−x_Si_x_O_4_ (x = 0, 0.025, 0.05, 0.075 and 0.10) samples. The reversible capacity and cycling life of the LiMn_2−x_Si_x_O_4_ (x = 0, 0.025, 0.05) samples were remarkably enhanced as the silicon doping content increased, due to the more regular and increased MnO_6_ octahedra, which is conducive to the lithium ion diffusion in the electrochemical redox process [24]. However, it should be noted that the introduction of more silicon ions has great negative impact on the reversible capacities of the LiMn_2−x_Si_x_O_4_ (x = 0.075, 0.10) samples despite the improvement of cycling life (Figure 5c). These unsatisfying results are principally because introducing more silicon ions can cause a reduction in the tetravalent manganese ions, which is unfavorable to Mn(III)−Mn(IV) interconversion. When the silicon doping content is 0.075 and 0.10, the adverse effect exceeds the positive influence from the more regular and increased MnO_6_ octahedra. Therefore, the electrochemical performance the LiMn_2−x_Si_x_O_4_ (x = 0.075, 0.10) samples will deteriorate to some extent.

Figure 5d presents the long cycling life of the LiMn_2−x_Si_x_O_4_ (x = 0, 0.025, 0.05 and 0.075) samples. For the LiMn_1.95_Si_0.05_O_4_ sample, the reversible capacity peaked at 140.8 mAh g^−1^, which is higher than that of the LiMn_2−x_Si_x_O_4_ (x = 0, 0.025 and 0.075) samples. Even more importantly, the LiMn_1.95_Si_0.05_O_4_ sample exhibited 128.3 mAh g^−1^ after 100 cycles, with an outstanding retention of 91.1%. Unfortunately, the LiMn_2−x_Si_x_O_4_ (x = 0, 0.025 and 0.075) samples showed a lower capacity with worse cycling life. In particular, the undoped spinel only delivered 132.7 mAh g^−1^ with low retention of 62.5% after 100 cycles. These analyses indicate that the introduction of silicon ions dramatically enhances the electrochemical performance of LiMn_2_O_4_.

**Figure 6:**
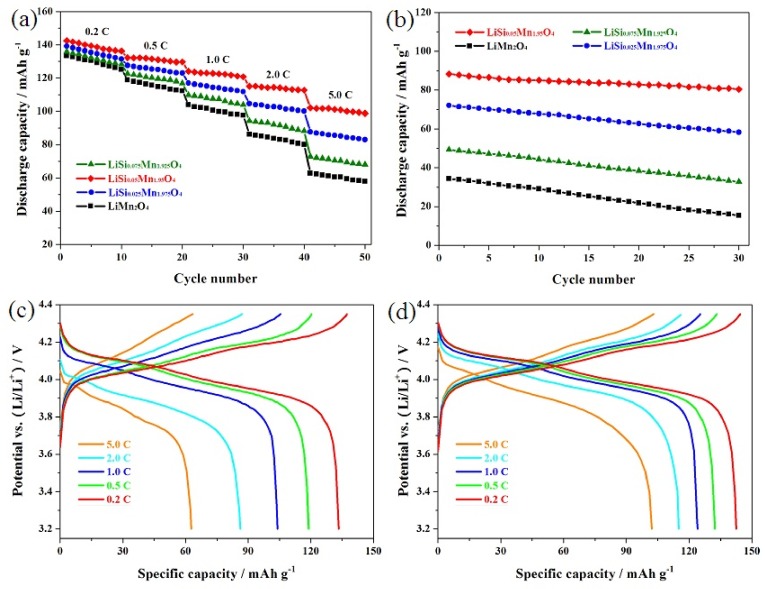
(**a**) Rate performance of the LiSi_x_Mn_2−x_O_4_ (x = 0, 0.025, 0.05, 0.075) samples; (**b**) Cycling performance of the LiSi_x_Mn_2−x_O_4_ (x = 0, 0.025, 0.05, 0.075) samples at the higher discharge rate of 10 C; Representative charge-discharge curves of the LiMn_2_O_4_ (**c**) and LiSi_0.05_Mn_1.95_O_4_ (**d**) samples at varying rates of 0.2–5.0 C.

Figure 6a shows the rate performance of the LiMn_2−x_Si_x_O_4_ (x = 0, 0.025, 0.05 and 0.075) samples. For all these samples, the increased rate has a great negative impact on the reversible capacity because the high rate seriously interferes with the diffusion process of lithium ions [26]. Among these samples, the LiMn_1.95_Si_0.05_O_4_ sample showed relatively good rate capability with that of the LiMn_2−x_Si_x_O_4_ (x = 0, 0.025 and 0.075) samples at a high rate. When cycled at 0.2 C, the capacities of the LiMn_2_O_4_, LiMn_1.975_Si_0.025_O_4_, LiMn_1.95_Si_0.05_O_4_ and LiMn_1.925_Si_0.075_O_4_ samples reached 133.4, 135.9, 139.2 and 142.5 mAh·g^−1^, respectively. However, it is important to note that the discharge capacities of these Si-doped samples show more and more obvious difference at 5.0 C. The LiMn_1.95_Si_0.05_O_4_ sample could show 102.1 mAh·g^−1^, while the LiMn_2_O_4_, LiMn_1.975_Si_0.025_O_4_ and LiMn_1.925_Si_0.075_O_4_ samples showed lower discharge capacities of 62.8, 72.8 and 87.7 mAh·g^−1^. The above discussion indicates that the optimal Si-doping amount can produce the best improvement effect on the electrochemical performance on the premise that all the silicon-doped samples involved a small amount of Si^4+^ ions.

To further explore the rate performance at high rates, the LiMn_2−x_Si_x_O_4_ (x = 0, 0.025, 0.05 and 0.075) samples were tested at 10 C, and the corresponding test results are presented in Figure 6b. For the LiSi_0.05_Mn_1.95_O_4_ sample, the reversible capacity of the first cycle could exhibit 88.3 mAh·g^−1^, which is much higher than that of the LiMn_2_O_4_, LiMn_1.975_Si_0.025_O_4_ and LiMn_1.925_Si_0.075_O_4_ samples. Moreover, the LiMn_1.95_Si_0.05_O_4_ sample showed a satisfactory reversible capacity of 80.4 mAh·g^−1^ after 30 cycles with an outstanding retention of 91.2%. For the LiMn_2−x_Si_x_O_4_ (x = 0, 0.025 and 0.075) samples, a lower reversible capacity with worse cycling stability was presented. The above results further confirmed that the best improvement effect was obtained by introducing an optimal amount of Si^4+^ ions.

Figure 6c,d present the representative discharge curves of the undoped LiMn_2_O_4_ and the LiMn_1.95_Si_0.05_O_4_ samples at varying rates. As shown here, there are two obvious voltage platforms at 0.2 C and 0.5 C, suggesting the diffusion process of lithium ions [33]. When the rate was further increased, these two potential plateaus gradually show ambiguous boundaries and shift toward lower voltage as the discharge rate increases. This result has a lot to do with the ohmic drop and the polarization effect [9]. Compared with the LiMn_1.95_Si_0.05_O_4_ sample, the undoped LiMn_2_O_4_ sample showed a lower platform at high rate and a more obvious reduction in capacity. The above analysis indicates that the introduction of some silicon ions can have a positive effect on high rate performance.

**Figure 7:**
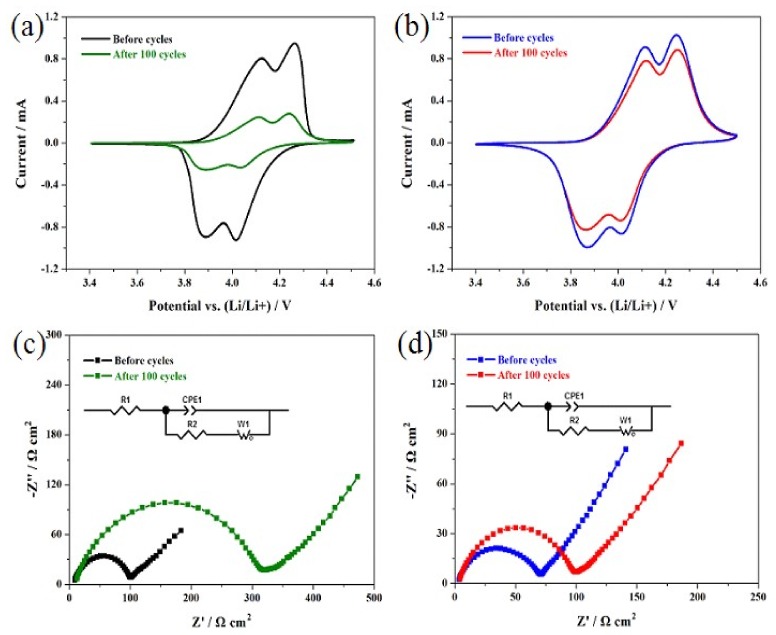
Cyclic voltammogram curves of the LiMn_2_O_4_ (**a**) and LiSi_0.05_Mn_1.95_O_4_ (**b**) samples at a scan rate of 0.15 mV s^−1^; Nyquist plots of the LiMn_2_O_4_ (**c**) and LiSi_0.05_Mn_1.95_O_4_ (**d**) samples before cycling and after 100 cycles.

Figure 7a,b show the cyclic voltammogram results of the undoped LiMn_2_O_4_ and the LiMn_1.95_Si_0.05_O_4_ samples. As shown in Figure 7a, the undoped LiMn_2_O_4_ possesses two pairs of redox peaks, which correspond to the relevant diffusion process of lithium ions [34]. It is important to note that the redox peak current decreased significantly after 100 cycles, suggesting that the undoped LiMn_2_O_4_ sample did not show outstanding cycling performance [35]. Figure 7b presents the results of the LiMn_1.95_Si_0.05_O_4_ sample. We can see that there are few significant changes in the peak currents. These results suggest that the introduction of some silicon ions plays an effective role in enhancing lithium ion diffusion.

Figure 7c,d show the Nyquist plots of the undoped LiMn_2_O_4_ and the LiMn_1.95_Si_0.05_O_4_ samples. According to the reported results [9,19], the charge transfer resistance (R_2_) corresponding to the high-frequency semicircle has much to do with cycling life. Therefore, the influence of introducing some silicon ions on the cycling life was studied by a thorough analysis of R_2_ values. Table 2 lists the relevant fitting values of R_2_. For the Si-doped spinel, the original R_2_ value only reached 61.5 Ω cm^2^ and increased to 90.6 Ω cm^2^ with a low growth rate of 47.3% after 100 cycles. Compared with the Si-doped spinel, the undoped spinel presents a higher original R_2_ value (92.3 Ω·cm^2^). After 100 cycles, this value could reach up to 302.7 Ω·cm^2^ with a very high growth rate of 228.0%. These analyses indicate that introducing some silicon ions can help to decrease the R_2_ value, which can promote lithium ion diffusion [18].

## 4. Conclusions

Silicon-doped lithium manganese oxides were obtained via a sol-gel process. As the optimal Si-doped spinel, the LiSi_0.05_Mn_1.95_O_4_ sample possessed a regular surface morphology and an even size distribution. More importantly, it showed much better electrochemical properties than those of the other Si-doped LiMn_2_O_4_ samples with a small amount of Si^4+^ ions. When cycled at 0.2 °C and 0.5 °C, the LiMn_1.95_Si_0.05_O_4_ sample exhibited 142.5 and 140.8 mAh·g^−1^, respectively, which are higher values than those of the LiMn_2_O_4_, LiMn_1.975_Si_0.025_O_4_ and LiMn_1.925_Si_0.075_O_4_ samples. After 100 cycles, the LiMn_1.95_Si_0.05_O_4_ sample could exhibit 128.3 mAh·g^−1^ with an outstanding retention of 91.1% at 0.5 °C. When cycled at 10 °C, the initial discharge capacity of the optimal Si-doped LiMn_2_O_4_ sample could exhibit 88.3 mAh·g^−1^. All of these results suggest that the optimal Si-doping amount can produce the best improvement effect on the electrochemical performance on the premise that all the silicon-doped spinels involved a small amount of Si^4+^ ions.

## Figures and Tables

**Table 1:** Crystal parameters of the LiSi_x_Mn_2−x_O_4_ (x = 0, 0.025, 0.05, 0.075 and 0.10) samples.

Sample	Space	a (nm)	Volume (nm^3^)	*I*_311_/*I*_400_	FWHM_400_
LiMn_2_O_4_	Fd-3m	0.82325	0.55795	0.8992	0.291
LiSi_0.025_Mn_1.975_O_4_	Fd-3m	0.82328	0.55801	0.9274	0.278
LiSi_0.05_Mn_1.95_O_4_	Fd-3m	0.82335	0.55815	0.9645	0.243
LiSi_0.075_Mn_1.925_O_4_	Fd-3m	0.82344	0.55834	0.9587	0.258
LiSi_0.10_Mn_1.90_O_4_	Fd-3m	0.82360	0.55866	0.9453	0.265

**Table 2:** Fitting values of the charge transfer resistance (R_2_) calculated from EIS.

Sample	R_2_ (Ω·cm^2^) Before Cycles	R_2_ (Ω·cm^2^) After 100 Cycles	Percentage of Increase
LiMn_2_O_4_	92.3	302.7	228.0%
LiSi_0.05_Mn_1.95_O_4_	61.5	90.6	47.3%
